# P-522. PrEP Decision Factors: a CHORUS Healthcare Professional Survey

**DOI:** 10.1093/ofid/ofae631.721

**Published:** 2025-01-29

**Authors:** Ricky K Hsu, Kevin R Frost, Brooke Levis, Rachel P Weber, Carl Millner, Steven K Barnett, Courtney Sherman, Cindy Markarian, Jennifer S Fusco, Michael D Osterman, Supriya Sarkar, Vani Vannappagari, Jean A van Wyk, Gregory P Fusco

**Affiliations:** AIDS Healthcare Foundation/ NYU School of Medicine, New York, New York; amfAR, The Foundation for AIDS Research, New York, New York; Epividian, Inc, Montreal, Quebec, Canada; Epividian, Inc., Raleigh, North Carolina; AIDS Healthcare Foundation, Los Angeles, California; CAN Community Health, Sarasota, Florida; CAN Community Health, Sarasota, Florida; AIDS Healthcare Foundation, Los Angeles, California; Epividian, Inc., Raleigh, North Carolina; Epividian, Inc., Raleigh, North Carolina; ViiV Healthcare, Baltimore, Maryland; ViiV Healthcare, Baltimore, Maryland; ViiV Healthcare, Brentford, UK, Brentford, England, United Kingdom; Epividian, Inc., Raleigh, North Carolina

## Abstract

**Background:**

Pre-exposure prophylaxis (PrEP) has been available since 2012, yet only 3 in 10 individuals at increased risk of human immunodeficiency virus (HIV) acquisition are currently prescribed PrEP in the US. We assessed factors related to PrEP prescription by healthcare professionals (HCPs).

**Figure:**
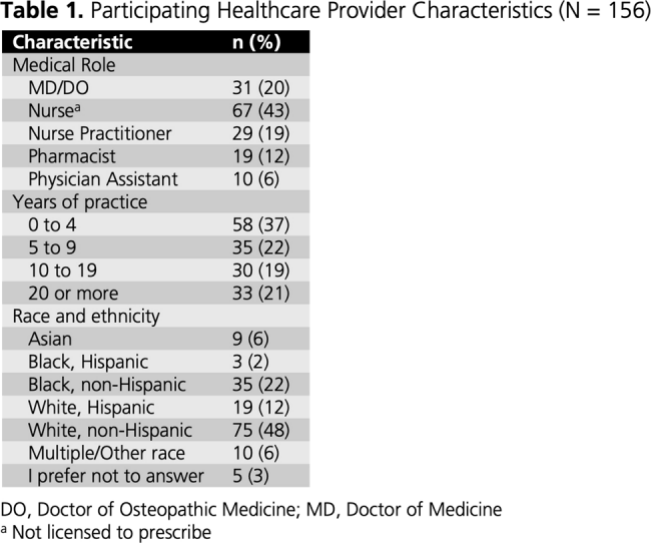

**Methods:**

HCPs that use the CHORUS phone application were invited to a survey including 50 multiple-choice questions related to PrEP prescribing. Responses were summarized.

**Figure:**
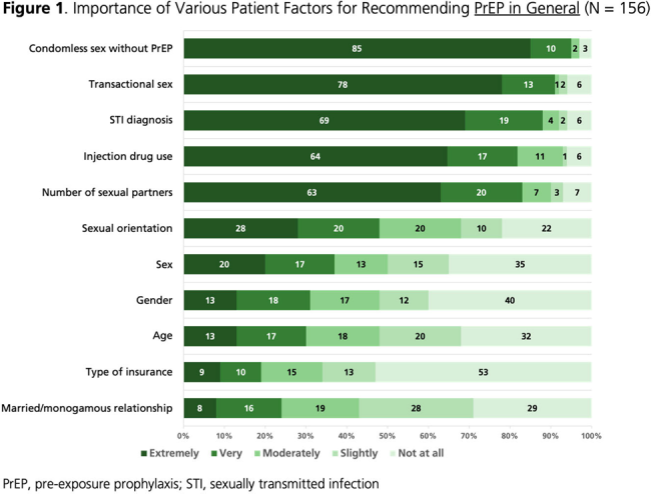

**Results:**

From 04OCT2023 to 22MAR2024, 156 HCPs completed the survey (**Table 1**); 59% were cisgender women. Self-reported PrEP knowledge was high (86% very good/excellent; 14% good). Almost all (99%) perceived PrEP as very/extremely effective in preventing HIV acquisition. More than half (57%) perceived long-acting (LA) PrEP as more effective than oral PrEP, while 40% perceived similar effectiveness. Most (87%) believed it is important to offer PrEP in addition to condoms. HCPs rated condomless sex without PrEP, transactional sex, and STI diagnoses as the most important factors for recommending PrEP (**Fig. 1-2**). Most (88%) indicated they often/always initiate conversations about PrEP with sexually active patients. Conversely, 45% are often/always asked about PrEP in general, and 28% are often/always asked about LA PrEP. Among 89 HCPs licensed to prescribe, all reported being very/extremely likely to prescribe PrEP to individuals at high risk of HIV acquisition; the percentage was 63% for prescribing LA PrEP specifically. HCPs indicated that they were most likely to prescribe LA PrEP to individuals who have condomless sex with men of unknown HIV status or have multiple sex partners, as well as transgender individuals who have sex with men (**Fig. 3**).

**Figure:**
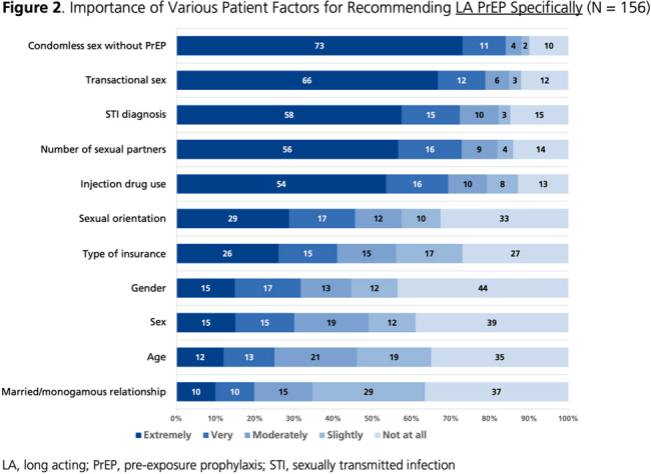

**Conclusion:**

HCPs expressed knowledge and positive perceptions about PrEP in general and LA PrEP specifically as well as willingness to consider PrEP for individuals at risk of HIV acquisition. Almost half do not view LA PrEP as more effective than oral PrEP, despite clinical trial evidence to the contrary. Most initiate conversations about PrEP with their patients but less than half are asked about PrEP by their patients and even fewer are asked about LA PrEP specifically. Work is needed to increase HCP knowledge of LA PrEP’s efficacy in and suitability for various populations in need of HIV prevention.

**Figure:**
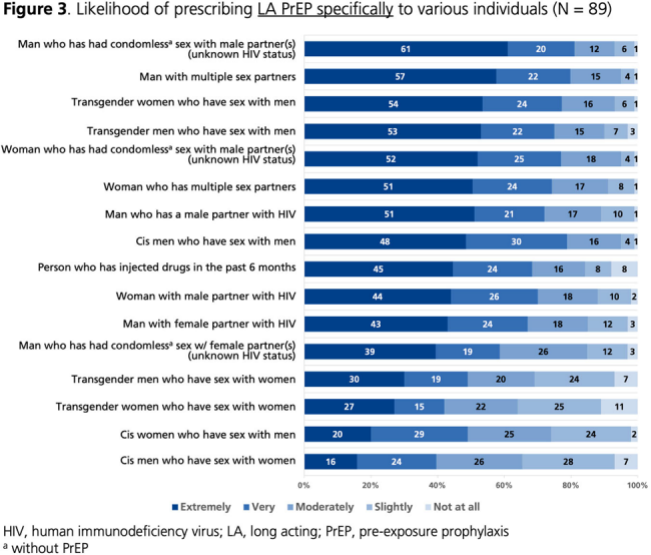

**Disclosures:**

**Ricky K. Hsu, MD**, EMD Serono: Advisor/Consultant|EMD Serono: Honoraria|Gilead: Grant/Research Support|Gilead: Honoraria|Merck: Honoraria|ViiV: Advisor/Consultant|ViiV: Grant/Research Support|ViiV: Honoraria **Brooke Levis, PhD**, EMD Serono: Research support to my employer|Gilead Sciences: Research support to my employer|Merck & Co.: Research support to my employer|TheraTechnologies: Research support to my employer|ViiV Healthcare: Research support to my employer **Rachel P. Weber, PhD**, EMD Serono: Research support to my employer|Gilead Sciences: Research support to my employer|Merck & Co.: Research support to my employer|TheraTechnologies: Research support to my employer|ViiV Healthcare: Research support to my employer **Jennifer S. Fusco, BS**, EMD Serono: Research support to my employer|Gilead Sciences: Research support to my employer|Merck & Co.: Research support to my employer|TheraTechnologies: Research support to my employer|ViiV Healthcare: Research support to my employer **Michael D. Osterman, PhD**, EMD Serono: Research support to my employer|Gilead Sciences: Research support to my employer|Merck & Co.: Research support to my employer|TheraTechnologies: Research support to my employer|ViiV Healthcare: Research support to my employer **Supriya Sarkar, PhD, MPH**, GSK: Stocks/Bonds (Public Company)|ViiV Healthcare: Full time employee **Vani Vannappagari, MBBS, MPH, PhD**, GSK: Stocks/Bonds (Public Company)|ViiV Healthcare: Full time Employee|ViiV Healthcare: Stocks/Bonds (Public Company) **Jean A. van Wyk, MBChB, MFPM**, ViiV Healthcare: Employee|ViiV Healthcare: Stocks/Bonds (Public Company) **Gregory P. Fusco, MD, MPH**, EMD Serono: Research support to my employer|Gilead Sciences: Research support to my employer|Merck & Co.: Research support to my employer|TheraTechnologies: Research support to my employer|ViiV Healthcare: Research support to my employer

